# Numerical Analysis of the Cell Droplet Loading Process in Cell Printing

**DOI:** 10.3390/mi15111335

**Published:** 2024-10-31

**Authors:** Yankun Wang, Fagui Pang, Shushan Lai, Renye Cai, Chenxiang Lai, Zexin Yu, Yiwei Zhu, Min Wu, Heng Zhang, Chunyu Kong

**Affiliations:** 1 School of Automobile and Transportation Engineering, Guangdong Polytechnic Normal University, Guangzhou 510665, China; wyk@gpnu.edu.cn (Y.W.); pangfagui@163.com (F.P.); laishushan2003@163.com (S.L.); yiweizhu@gpnu.edu.cn (Y.Z.); wumin35045@126.com (M.W.); zhangh08200@126.com (H.Z.); 2 Guangzhou Metro Design & Research Institute Co., Ltd., Guangzhou 510010, China; 3 Institute for Manufacturing Technologies of Ceramic Components and Composites (IMTCCC), University of Stuttgart, Allmandring 7b, 70569 Stuttgart, Germany; yu.zexin@ifkb.uni-stuttgart.de

**Keywords:** cell printing, impact, cell damage, finite element method (FEM)

## Abstract

Cell printing is a promising technology in tissue engineering, with which the complex three-dimensional tissue constructs can be formed by sequentially printing the cells layer by layer. Though some cell printing experiments with commercial inkjet printers show the possibility of this idea, there are some problems, such as cell damage due the mechanical impact during cell direct writing, which include two processes of cell ejection and cell landing. Cell damage observed during the bioprinting process is often simply attributed to interactions between cells and substrate. However, in reality, cell damage can also arise from complex mechanical effects caused by collisions between cell droplets during continuous printing processes. The objective of this research is to numerically simulate the collision effects between continuously printed cell droplets within the bioprinting process, with a particular focus on analyzing the consequent cell droplet deformation and stress distribution. The influence of gravity force was ignored, cell droplet landing was divided into four phases, the first phase is cell droplet free falling at a certain velocity; the second phase is the collision between the descending cell droplet and the pre-existing cell droplets that have been previously printed onto the substrate. This collision results in significant deformation of the cell membranes of both cell droplets in contact; the third phase is the cell droplet hitting a rigid body substrate; the fourth phase is the cell droplet being bounced. We conducted a qualitative analysis of the stress and strain of cell droplets during the cell printing process to evaluate the influence of different parameters on the printing effect. The results indicate that an increase in jet velocity leads to an increase in stress on cell droplets, thereby increasing the probability of cell damage. Adding cell droplet layers on the substrate can effectively reduce the impact force caused by collisions. Smaller droplets are more susceptible to rupture at higher velocities. These findings provide a scientific basis for optimizing cell printing parameters.

## 1. Introduction

In recent years, three-dimensional (3D) printing technology has become the focus in tissue engineering and regenerative medicine. When applied to biological scaffolds, 3D printing enables more accurate geometries with sufficient mechanical strength, so it is often widely used in tissue engineering [1,2]. Cell printing is based on the following principle of developmental biology: cells self-assembly into tissues. This is similar to the way embryonic-like tissues fuse into functional forms. By sequentially printing the cells layer by layer, three-dimensional tissue constructs can be formed. The latest development in cell printing is using tissue regeneration 3D technology to print more complex organs, which still requires a lot of effort to research [3]. Tissue regeneration 3D technology is the use of 3D printing technology to construct three-dimensional tissue and organ structures. Integral to this technology is using biocompatible materials and bio inks, which are usually hydrogels containing living cells. These materials are designed to support cell growth, proliferation, and differentiation, enabling the acquisition of multiple cells of the same type for repair or replacement of dead or damaged cells, thereby forming new tissues [4]. Therefore, studying the stress distribution and activity maintenance of cells during continuous printing is crucial to ensuring that the printed structures have biological activity and functionality.

There are a lot of experiments on cell printing, including printing various cell droplets on biological bases to build patterns, and printing cells in specific organs to study their regenerative functions. Various printing technologies already exist, such as inkjet printing [5], extrusion printing [6], and laser-assisted printing [3]. Sun et al. printed self-assembled I3QGK peptide nanofibers for a silk-based substrate using an inkjet printer, forming a complex patterns [7]. Lin et al. printed MCF-7 cells on a hydrophobic substrate by using an inkjet printer [8]. Kim et al. printed a full thickness 3D human skin model and a pre-vascularized skin patch by coming extrusion and inkjet printing techniques [9]. Subsequently, the same team also printed high-density cancer cells with different diameters using 3D printing technology [10]. Singh et al. printed vascularized renal proximal tubules using 3D printing technology [11]. Chae et al. printed fibrocartilaginous tendon–bone interfaces (TBIs) to study rotator cuff regeneration [12]. Yang et al. built a micro-patterned cell-repellent interface through burning the polydimethylsiloxane (PDMS)-based substrate using a femtosecond-laser direct writing system [13]. Cell printing techniques have been employed successfully by precisely positing different biological materials to form different compositions of design and structure. The long-term possibility of using this new technology could be tailoring tissue-engineered regenerative therapies.

In the field of cell printing, the distribution of forces acting on cells during the printing process and the resulting damage have always been the focus of research. Research is usually conducted through a combination of experimental methods and modeling techniques. Lin et al. observed that the viability of HT-29 cells decreased from 95% to 78% as the laser fluence increased from 258 to 1482 mJ/cm^2^, and noted that cellular damage in this study was primarily attributed to the mechanical stress induced during cell droplet formation and landing processes, with thermal effects and ultraviolet radiation having a minor impact [14]. He et al. developed a fluid dynamics model based on multiphase fluid–structure interactions to reveal the dynamic behavior and stress distribution of cells during the printing process, providing a theoretical basis for optimizing cell viability [15]. Ladjal et al. developed a micro-to-nano biomechanical model incorporating real-time haptic and visual feedback to simulate the cell injection process, but did not fully account for liquid flow effects [16]. Blaeser et al. used a hydrodynamic model to develop formulas for studying the effect of shear stress on cell viability [17]. Ning et al. established a cell damage model based on experiments with a rheometer and calculated cell damage caused by shear stress and extension stress [18]. Huang et al. developed a cell printing experiment system utilizing a commercial piezoelectric inkjet printing head and a configurable drive circuit. They demonstrated that a large driving pulse width is beneficial for printing cell solutions and that placing soy agar gel on substrates helps to maintain the activity of the printed cells [19]. Ringeisen et al. discovered that enhancing the substrate hydrogel coating from 20 μm to 40 μm significantly boosted post-transfer cell viability, increasing it from 50% to 95% [20]. In both pneumatic and screw-driven mechanisms, the main cause of cell membrane rupture is process-induced forces such as hydrostatic pressure, shear stress, extensional stress, and tensile/compressive forces [21]. Han et al. used numerical calculations and experiments to understand the process of cell damage caused by forced extrusion bioprinters. A differential equation for the growth of the damaged cell ratio (DCR) was developed, and the proposed formula was able to effectively predict the measurements of the DCR under 43 bioprinting conditions [22].

Cell printing was divided into three processes, cell droplet formation, cell ejection and cell droplet landing. During these processes, cells are continually subjected to mechanical forces. Wang et al. found that the heat transfer and high pressure (typically ranging from 8 to 15 MPa) during landing can easily cause damage [23]. Simultaneously manipulating time is also a key parameter, like mechanical forces, that can affect the activity of printed cells. Manipulating time refers to the duration of ejection of cell droplets (i.e., bioink), which subsequently affects the total exposure time of cells to mechanical forces during the printing process, including parameters like the printer’s drive pulse width and firing interval. Cai et al. found that increasing the firing time interval can effectively reduce cell damage and maintain the activity of printed cells during the laser-assisted cell ejection process [24]. Huang et al. reported that yeast cells exhibited rather high viability post-printing when subjected to a driving pulse width ranging from 10 to 25 μs. Furthermore, an increased pulse width was observed to enhance the post-print cell suspension concentration and to mitigate nozzle clogging issues [19].

Modeling cell injury incurred in these processes is lacking in the literature, but it is known that Schwann cells and fibroblasts are commonly used to study the regenerative function of cells after injury [25]. In order to understand the actual cell damage, the finite element method is usually used to observe the transition of cell droplets under the collision force during cell landing, and the impact force of cell droplets and hydrogel contact collision is calculated [26]. It was found that cells in cell droplet formation and the cell ejection process would also be hurt. This paper ignores these effects and mainly researches cell damage during cell landing. In practice, the cell was printed layer by layer. The cell droplet was directed towards contact with other cell droplets. Therefore, a model of impact between cell droplets was set up in this study. The influence of cell droplet collision velocity and the number of cell layers on the substrate on cell velocity, cell acceleration and pressure status is studied to achieve a better understanding of cell damage during cell landing.

## 2. Computational Procedures

### 2.1. Process Governing Equations

**Figure 1:**
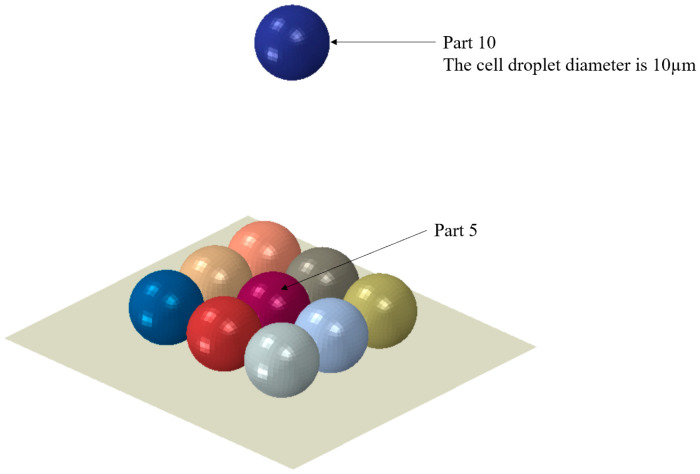
Cell droplet diagram.

Cell damage during the landing process is an unavoidable problem in generic cell printing processes, whether laser assisted or inkjet based. A short-form diagram of one of the cell droplets during cell droplet collision is shown in Figure 1. When a cell droplet falls with vertical velocity after it was ejected from a supporting medium, it travels a certain distance and hits the other cell droplets that have been printed on the substrate. Eventually, the cell droplet reaches the rigid substrate if the terms (cell droplet collision velocity and the number of cell layers on the substrate) satisfy a certain condition, and then rebounds back out. This study assumes the model is not affected by gravity and the cell droplet is made of a single material. The falling cell droplet and the printed cell droplet have the same mass and chemical properties.

During the cell landing process, the cell droplet takes on a remarkable transformation to survive a much higher external force than it is capable of under steady-state conditions. This contact–impact process can be modeled by governing equations, such as the momentum equation, which is a form of the Navier–Stokes equation [27]:

(1) $$\sigma_{ij}+\rho f_{j}=\rho \ddot{x}$$

Here, $\sigma_{ij}$ represents the Cauchy stress tensor, which encompasses both the viscous stresses arising from the fluid’s internal friction and the stresses resulting from pressure gradients within the fluid. The symbol ρ in the Equation (1) is the density of the fluid, $f_{j}$ is the body force per unit mass, and $\ddot{x}$ is the acceleration of the fluid. In practical applications, this equation usually needs to be solved together with the continuity equation (mass conservation) and energy equation (energy conservation) of the fluid to obtain the distribution of the fluid velocity field, the pressure field, and other related physical quantities.

The mass conservation is trivially stated [27]:

(2) $$\rho V=\rho_{0}$$

This equation states that the density ρ of a fluid element times its volume V is constant and equal to a reference density $\rho_{0}$. This is a simplification that assumes the fluid is incompressible.

The energy equation is represented as [27]:

(3) $$\dot{E}\;={\;Vs}_{ij}\dot{\varepsilon_{ij}}-(p+q)\dot{V}$$

where $\dot{E}$ is the rate of change in energy in a fluid element. The term ${Vs}_{ij}\dot{\varepsilon_{ij}}$ represents the work done due to the viscous forces inside the fluid, where $s_{ij}$ is the partial stress part of the stress tensor $\sigma_{ij}$, which is the remaining part after subtracting the pressure p from the total stress. The deviatoric stress tensor describes the frictional or viscous forces inside a fluid. And $\dot{\varepsilon_{ij}}$ is the strain rate tensor, which represents the rate of fluid deformation. The item (p + q)$\dot{V}\;$ represents the energy changes caused by pressure p and heat flux q. $\dot{V}$ is the rate of change in the volume of fluid elements.

This equation is integrated over time for the evaluation of the state equation and the calculation of global energy balance. To meet the traction boundary conditions, we have:

(4) $$\sigma_{ij}n_{i}=t_{i}(t)$$

where $n_{i}$ represents the unit outward normal to the boundary element, and $t_{i}$(*t*) denotes the traction vector acting on the boundary, which is a function of time t.

In addition, the determinant $F_{ij}$ of the deformation gradient matrix is defined as:

(5) $$F_{ij}=\frac{\partial_{x_{i}}}{\partial_{X_{i}}}$$

where $x_{i}$ are the coordinates in the current configuration and $X_{i}$ are the coordinates in the reference configuration.

### 2.2. Contact Algorithms

In this study, a simulation model of cell droplet collision was established by using ABAQUS 2020 software to investigate the mechanism of cell damage. ABAQUS has implemented a variety of contact algorithms: the Lagrange multiplier method, the penalty function method, etc. The use of the normal Lagrangian method can result in contact disturbances, as the contact state is a step function, making it impossible to determine whether the contact state is open or closed, which can sometimes make convergence more difficult [28], while the penalty function method has the characteristics of efficient computational performance and easy implementation. Therefore, to simulate this nonlinear dynamic process, the penalty function method was adopted to handle contact constraints. This method approximates the contact behavior by introducing virtual springs between the contact surfaces, effectively avoiding penetration of the contact surfaces [29]. The mathematical expression of the penalty function method can be expressed as:

(6) $$F_{normal}=k_{normal}X_{penetration}$$

where $F_{normal}$ is the contact force (Normal Force), $k_{normal}$ is the penalty stiffness, and $X_{penetration}$ is the penetration between the contact surfaces. The penalty stiffness $K_{n}$ is a very large value, usually automatically selected by Abaqus.

### 2.3. Material Models

This paper mainly studies the impaction between cell droplet and cell droplet. Both cells are the same kind of cells, which have the same mass and chemical properties. The size of the cell droplets is indeed influenced by the printer nozzle. Therefore, a one-material model is utilized within the computational domain to simulate the cell droplet.

#### The Cell Droplet

In recent years, studies on the mechanical model of living cells have made notable progress. Due to the complex structure and diverse composition of living cells, numerous mechanical models have been developed by researchers employing both micro/nanostructural and continuum approaches [30]. During the cell droplet landing process, both the falling cell droplet and the printed cell droplet are undergoing a relatively severe deformation and impaction, especially the cell droplet that in the crush zone that is subjected to tremendous mechanical stimulations. A linear elastic solid model was selected to model cell droplets in this study, to provide details on the distribution of stresses and strains on the cell to obtain a better understanding of cell damage during the printing process [24,31]. The linear elastic model is a simplification of the viscoelastic model, where the time factor has been neglected. A linear elastic material is described by [32]:

(7) $$\tau_{ij}=G\gamma_{ij}$$

where G is the shear modulus and is related to Young’s modulus E by E = 2(1 + v) G with v being Poisson’s ratio.$\tau_{ij}$ is the shear stress component and $\gamma_{ij}$ is the corresponding shear strain component. The cell droplet diameter was assumed to be 10 μm [33], density was assumed to be 1000 kg/m^3^, Young’s modulus E was set at 1.79 MPa [34], and Poisson’s ratio v was 0.475 [24].

## 3. Simulation Setup and Result

### 3.1. FEM Model Setup

During the process of research, simplified models have been proposed to make computation feasible. In this study, pre-printed cell droplets were idealized, assuming they were successfully printed perfectly. This simplified model helps to reduce the complexity of the model. The experimental results we conducted earlier showed that when only a few cell drops were printed on soy agar gel substrate, the cell survival rate could reach 96% [19]. This suggests that the initial layers of cell droplets can be deposited with minimal stress. When the first or second layer of cell droplets falls, there is a large amount of suspension on the substrate, which can effectively buffer the impact force generated when the cell droplets fall. As the number of printed cell droplets increases, subsequent cell damage mainly originates from the interactions between cell droplets. In order to focus on the collision effects between cell droplets during the cell printing process, we assume that the bottom layer of cell droplets is perfectly printed, which can effectively isolate variables and more accurately quantify the interactions between cell droplets. As shown in Figure 1, we have developed a simulation model illustrating the interaction between a freely falling cell droplet and a layer of pre-printed cell droplets. The model represents a system comprising ten droplets, designated as part 1 through part 10. In the simulation experiment, nine of these droplets were deposited onto a rigid substrate, and were arranged in a predetermined pattern to form a square array. A cell droplet (part 10) is descending towards the center of the substrate at a certain speed and is about to collide with the cell droplet (part 5) at the center of the substrate.

In this study, different predefined field velocities are applied to the descending balls and a fully fixed constraint is applied to the rigid bottom plate. In ABAQUS, the Universal Contact option provides a sophisticated way to ensure contact between a rigid surface and a deformable body (cell droplet) node. The substrate is treatment when stationary. The treated cell droplet was supported by a rigid substrate. The schematic of the grid model built is shown in Figure 1. A total of 36,896 nodes, 32,436 elements, and 10 parts were used to represent the model, which uses hexahedral mesh elements to divide the mesh and discretize the isometric mesh.

### 3.2. Representative Simulation Results

#### 3.2.1. Evolution of the Landing Process

**Figure 2:**
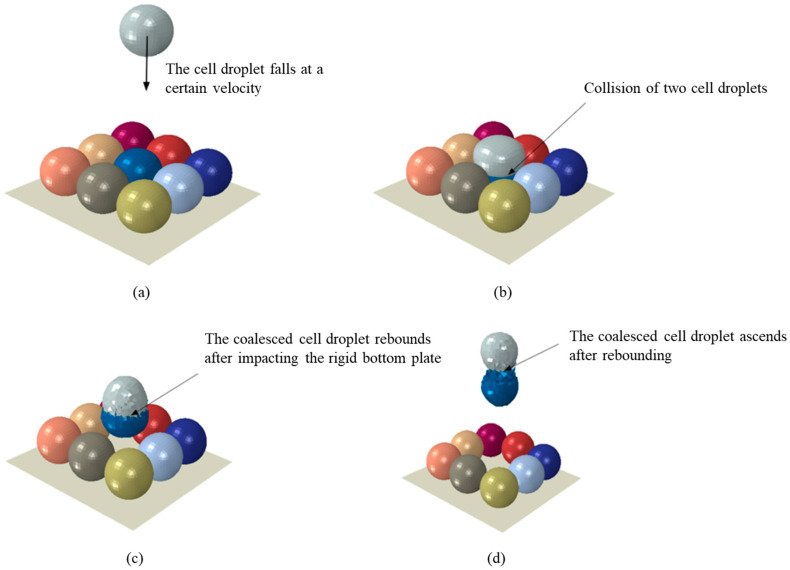
The landing process at (**a**) 0 us, (**b**) 0.26 μs, (**c**) 2.89 μs and (**d**) 3.67 μs.

In this paper, we will investigate the degree of damage caused by cell droplets hitting printed cell droplets supported by rigid substrates under different conditions. The key point of this study is the cell mechanical loading profile in the cell–cell landing process. Figure 2 shows the evolution of the landing process, and the influence of gravity force and friction are ignored. From Figure 2, it can be seen that cell landing was divided into four phases: the first phase is the cell droplet free falling at a certain velocity; the second phase is the impact between the cell droplet and the substrate cell droplet, which results in a relatively severe deformation of the cell membrane of both cell droplets in contact; the third phase is the cell droplet hitting a rigid body substrate; the fourth phase is the cell droplet being bounced.

**Figure 3:**
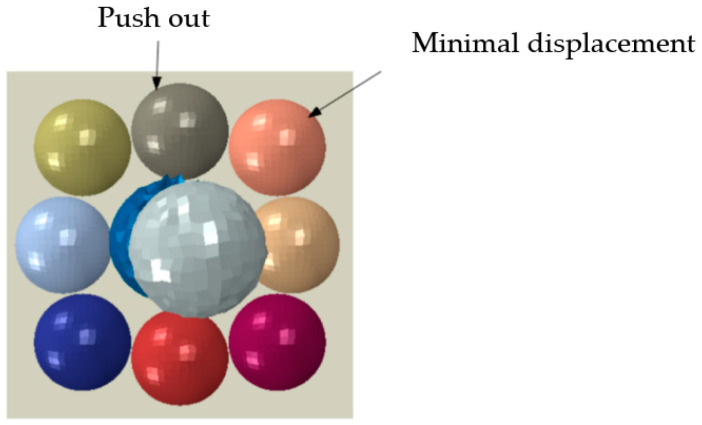
The phenomenon of intermediate and diagonal cells.

In the process, a cell droplet falls with normal velocity, which is opposite the middle cell droplet, and the two cell droplets undergo a transformation under the force of the collision and fuse together. The influence of the transformation of the two cell droplets is obvious. From Figure 3, four cell droplets near the middle cell droplet are ejected. The syncretic cell droplet is bounced at a certain velocity after hitting the rigid body substrate. Compared to other cell droplets on the substrate, the displacement of the four cell droplets on the diagonal cells is relatively small.

#### 3.2.2. The Effect of Different Jet Velocities

**Figure 4:**
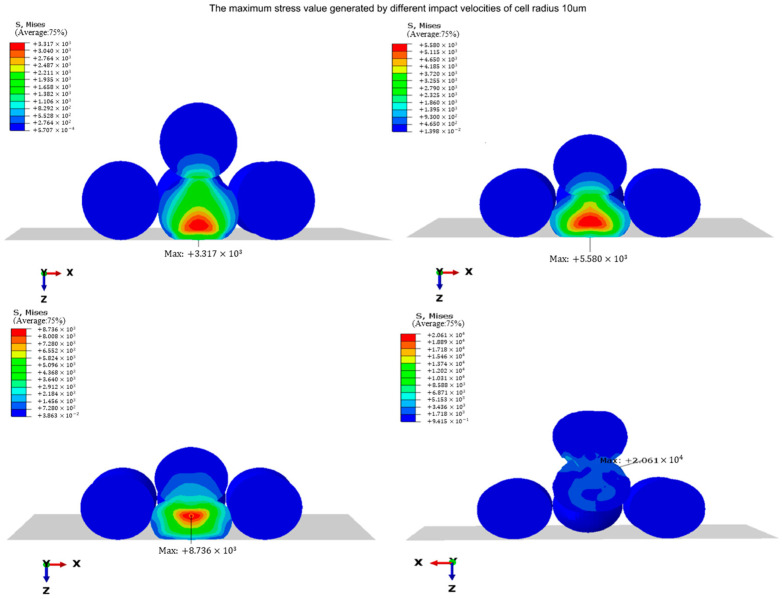
The maximum stress (Mpa) generated at different impact velocities.

In order to investigate the impact of varying jet velocities on the stress and strain experienced by cell droplets during the landing process, it is import to take into account that the cell droplets in the middle of the extrusion zone and the falling cell droplets undergo the most severe deformation, which means that this part is the most prone to cell damage. Therefore, these cell droplets (part 5 and part 10) were selected as research subjects in order to better understand the overall response of the cell droplets during landing. The simulations were performed with a cell droplet diameter of D = 10 μm and four different initial jet velocities (V1 = 10 m/s, V2 = 20 m/s, V3 = 30 m/s, V4 = 40 m/s). Figure 4 depicts the moment when the stress value of cell droplets reaches a maximum at different landing speeds. The entire impact process includes two different types of impacts: impacts between descending cell droplets and the previously printed cell droplets on the substrate, and impacts between cell droplets and the rigid matrix. These two shocks occurred consecutively. From the figure, it can be seen that during the collision process, part 5 was the most affected by the impact. Part 5 experiences maximum stress values of 3.32 MPa, 5.58 MPa, 8.74 MPa, and 20.61 MPa, respectively.

Table 1 provides a detailed list of the maximum stress values, maximum strain values, and their corresponding growth rates for part 5 and part 10. This table illustrates the effect of different velocities on the stress and strain experienced by cell droplets. It can be seen that the different landing speeds of descending cell droplets have varying degrees of impact on both part 5 and part 10. Compared to part 5, part 10 has been less affected, and experiences maximum stress values of 2.41 MPa, 3.67 MPa, 5.11 MPa, and 16.7 MPa, respectively. From the maximum strain value in Table 1, it can be seen that the deformation effects of part 5 and part 10 cell droplets caused by impact were almost equal at low speed (10 m/s) and high speed (40 m/s), with 0.093 vs. 0.093 at low speed and 1.309 vs. 1.296 at high speed. Within a moderate speed range, the deformation of part 5 is still greater than that of part 10. This is consistent with the trend of the maximum stress value.

It can be seen from the table that when the impact velocity increases from 10 m/s to 20 m/s, the cell undergoes a sharp deformation, the stress undergoes a rapid nonlinear increase, the maximum stress and strain of the middle cell and the descending cell in the bottom plate increase by different amplitudes, the growth rate is greater than 50%, and when the impact velocity increases from 20 m/s to 30 m/s, compared with the previous velocity range, the growth rate of the maximum stress value and the maximum strain value of the middle cells and the descending cells in the bottom plate decreased to varying degrees, but they were still positive. When the impact velocity increases from 30 m/s to 40 m/s, the maximum growth rate of stress and strain increases sharply, the stress and deformation degree of the cell increases, and the internal structure of the cell is greatly deformed in this velocity range, which increases the risk of cell damage due to large deformation.

#### 3.2.3. The Effect of Different Numbers of Layers

**Figure 5:**
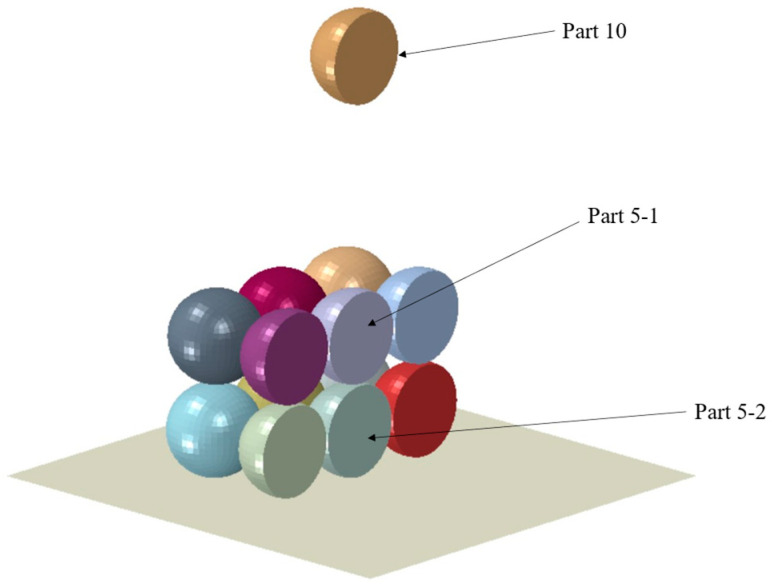
Schematic diagram of the simulation model.

**Figure 6:**
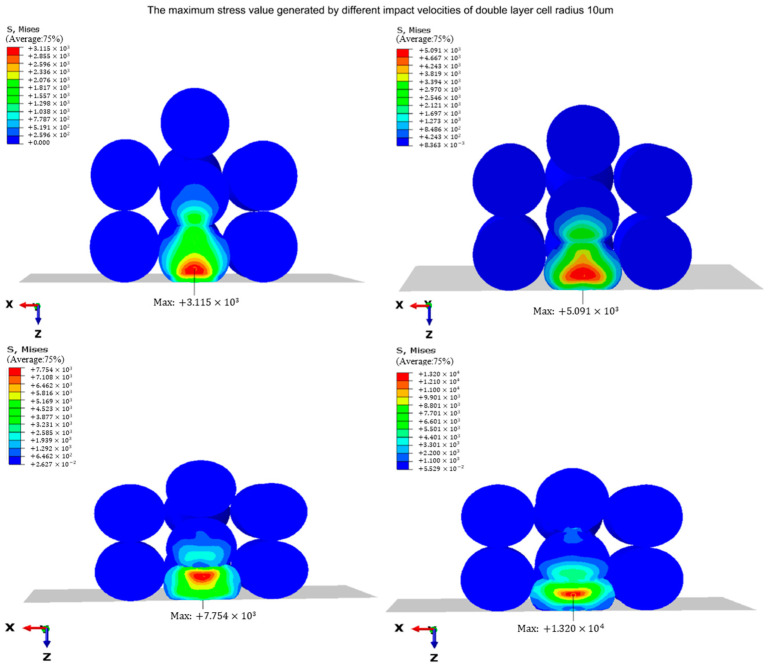
Maximum stress (Mpa) generated by double-layer cell droplets.

Cell printing is a complete three-dimensional structure layer by layer, and in order to study the damage caused by cell droplet collision, it is not enough to study the impact of descending cell droplets on a single layer of cell droplet—the effects of different layers must also be studied. The simulation was still carried out under the same conditions: a cell droplet diameter of D = 10 μm and a jet velocity of V = 10 m/s, 20 m/s, 30 m/s, and 40 m/s. The number of cell droplets on the substrate increased from 9 to 18, and they were placed in two layers, as shown in Figure 5. Figure 6 shows the stress contour of the cell droplet bouncing off against two layers of cell droplets at different velocities. Figure 6 was used to acquire the maximum stress value of the double-layer cell droplet impact.

From Table 1, it can be seen that when the speed is 10 m/s and a single-layer cell droplet is placed on the substrate, the maximum stress value of the cell droplet at the center of the bottom layer is 3.32 MPa. However, it can be observed from Table 2 that the maximum stress value of part 5_1 is 3.12 MPa when there is a double-layer cell droplet layer on the substrate. This suggests that the presence of an additional cell droplet layer can reduce the peak stress at the impact site. As the jet velocity increases to 20 m/s, 30 m/s, and 40 m/s, the maximum stress values for the bottom central droplet in a double-layer setup escalate to 5.09 MPa, 7.75 MPa, and 13.20 MPa, respectively. These values are consistently lower than those observed for a single cell droplet layer configuration, indicating that the greater the number of layers of the cell, the smaller the maximum stress value generated by the collision of cell droplets, the lower the risk of damage, and the greater the buffering effect of the number of layers.

More cell droplet layers will increase the cushioning effect. But it is also true that every cell droplet layer will impose its own stress upon cell landing and impact. Therefore, the accumulated stress on the bottom cell droplet may indeed be higher. This can be seen in Table 2—when compared to cell droplets in other layers, the total stress on the bottom cell droplets will be much higher. Comparing the maximum stress values of part 10 in Table 1 and part 10 in Table 2, it can be concluded that as the number of layers increases, the buffering effect on descending cell droplets is greater than that of the cell droplets at the bottom center. Observe the data in Table 1 and Table 2, where it was found that at medium and low speeds (10–30 m/s), the stress values of part 10 were relatively low, not exceeding 8 Mpa (cells are highly susceptible to damage under high-pressure (8–15 Mpa) conditions [23]), and the cells were able to maintain their survival ability. However, when the substrate is only coated with a single layer of cell droplets and the spraying speed reaches 40 m/s, the maximum stress in part 10 rises to 16.7 MPa. This exceeds the cell’s tolerance limit, leading to cell damage. When the jet velocity is 40 m/s, the maximum stress values of part 5 in Table 1 and part 5_1 in Table 2 are 13.20 Mpa and 20.61 Mpa, respectively, indicating that cell damage has occurred. On the contrary, increasing the number of cell droplet layers on the substrate to two resulted in a significant decrease in the maximum stress value of part 10 to 6.02 MPa. This reduction indicates that cells can maintain their activity under these conditions. At high speed, increasing the cell droplets of the substrate from one layer to two layers resulted in a significant decrease of 64% and 36% in the maximum stress values experienced by part 10 and part 5, respectively. This indirectly indicates that during the impact process, the increase in the number of layers has a greater buffering effect on the descending cell droplets, and each layer of droplets also increases the cumulative damage of the bottom center cell droplets. Optimizing the number of cell layers may be a key factor in reducing stress-induced cell damage and improving the overall success rate of bioprinting constructs.

#### 3.2.4. The Effect of Different Cell Droplet Diameters

During the landing process of cell droplets, the diameter of the ejected droplets can also cause changes in the stress and strain of the droplets. Therefore, we set the cell droplets to different diameter sizes D = 10 μm, 20 μm, and 30 μm and simulated their mechanical responses at different landing velocities V = 10 m/s, 20 m/s, 30 m/s, and 40 m/s. The model still simulates the interaction between a single-layer cell droplet layer and a falling cell droplet. Again, the same distorted cell droplets (Parts 5 and 10) were chosen as the research object.

**Figure 7:**
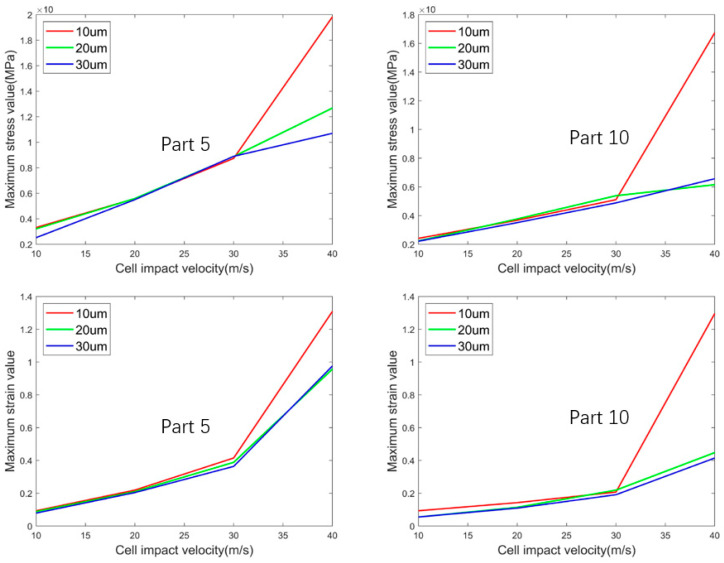
A line plot of the changes in the maximum stress and maximum strain values in parts 5 and 10 at different diameters and speeds.

Figure 7 shows a line plot of the changes in the maximum stress and maximum strain values in parts 5 and 10 at different diameters and speeds. The graph is made from the simulation data obtained from the simulation. Through the observation of Figure 7, it was found that when the cell droplet landing velocity is increased from 30 m/s to 40 m/s, the maximum stress and maximum strain values of the two cell droplets increase rapidly, which is not the same as that of the previous low velocity, and when the cell droplet impact velocity is less than 30 m/s, the maximum stress and strain values of the two cell droplets with different radii increase slowly. It can be seen that when the cell landing velocity is 30 m/s and below, the cell diameter sprayed by the printer nozzle is not the main factors affecting cell damage. However, when the cell landing velocity reaches 30 m/s or higher, cell droplets with a diameter of 10 µm experience greater stress and deformation compared to those with diameters of 20 µm and 30 µm, and are more susceptible to damage. Therefore, as the ejection velocity increases, there is a corresponding increase in the probability of cell damage in small-diameter cell droplets that are sprayed.

## 4. Conclusions

This study employed the finite element method to conduct an in-depth analysis of the landing behavior of cell droplets during the continuous printing process. The research identified four distinct stages of the landing process of cell droplets: the cell droplet free falling at a certain velocity; deformation due to impact between the falling cell droplet and the substrate cell droplet; the cell droplet hitting a rigid body substrate; and the fusion and rebound stage of cell droplets after impact. During this continuous process, the cell droplet on the substrate that is directly opposite and collides with the descending cell droplet was most significantly affected, characterized by considerable stress and substantial deformation.

The results of the simulation analysis revealed a positive correlation between the jet velocity and the stress on the cell droplets. As the spraying speed increases, the stress inside the cell droplets significantly increases, accompanied by more severe deformation, which increases the risk of cell damage. Specifically, when the spraying speed increases to 40 m/s, the stress on the cell droplets exceeds their tolerance threshold, leading to cell damage. Increasing the number of layers of cell droplets on the substrate can effectively reduce the impact force of collisions on cell droplets, thereby reducing the probability of cell damage. Especially under high-speed spraying conditions, increasing the number of layers of cell droplets on the substrate has a significant effect on alleviating the impact on descending cell droplets. In contrast, the buffering effect on the bottom layer cell droplets of the substrate is relatively limited. In the range of medium and low jet velocity (i.e., 10 m/s to 20 m/s), the diameter of cell droplets has no significant effect on the stress they bear. However, when the jet velocity is increased to 30 m/s or higher, smaller-diameter cell droplets will experience greater stress and more severe deformation, thereby increasing the risk of cell rupture. By finely regulating bioprinting parameters such as impact velocity and nozzle size, cell viability can be effectively maintained and cell damage during the printing process can be reduced. The research results provide a scientific basis for optimizing the design and printing parameters of bioprinting equipment.

## Figures and Tables

**Table 1:** Stress–strain values and their growth rates for cell droplet with four landing velocities.

	Cell Droplet at the Center of the Substrate (Part 5)				Descending Cell Droplet (Part 10)			
Velocity (m/s)	10	20	30	40	10	20	30	40
Stress maximum (MPa)	3.32	5.58	8.74	20.61	2.41	3.67	5.11	16.7
Maximum strain	0.093	0.219	0.415	1.309	0.093	0.143	0.207	1.296
growth rate of maximum stress	−	68.07%	56.63%	135.8%	−	52.28%	39.23%	226.8%
growth rate of maximum strain	−	134.7%	89.50%	215.4%	−	53.26%	44.76%	520.6%

**Table 2:** Maximum stress of cell droplet with four landing velocities (two-layer cell droplet on substrate).

Velocity (m/s)		10	20	30	40
Maximum Stress(MPa)	Part 10	2.31	3.58	4.65	6.02
	Part 5_2	2.43	4.11	6.11	6.70
	Part 5_1	3.12	5.09	7.75	13.20

## Data Availability

The original contributions presented in the study are included in the article, further inquiries can be directed to the corresponding authors.
